# Expert recommendations for *PIK3CA* testing in HR+/HER2− locally advanced and metastatic breast cancer

**DOI:** 10.1038/s41523-026-00968-3

**Published:** 2026-05-15

**Authors:** Caterina Marchiò, Federico Rojo, Ellen R. Copson, Fernando Schmitt, Beatriz Bellosillo, Carlo Fremd

**Affiliations:** 1 Pathology Unit, Candiolo Cancer Institute-FPO-IRCCS, Candiolo, Italy; 2 Department of Medical Sciences, University of Turin, Turin, Italy; 3 Pathology Department, IIS-Fundacion Jimenez Diaz University Hospital-CIBERONC-UAM, Madrid, Spain; 4 School of Cancer Sciences, Faculty of Medicine, University of Southampton, Southampton, UK; 5 RISE-Health, Department of Pathology, Medical Faculty of University of Porto, Porto, Portugal; 6 Pathology Department, Hospital del Mar Research Institute-CIBERONC-University Pompeu Fabra, Barcelona, Spain; 7 Department of Medical Oncology, National Center for Tumor Diseases (NCT), NCT Heidelberg, a partnership between DKFZ, the University Hospital Heidelberg (UKHD), the Heidelberg Medical Faculty of the Heidelberg University and the Thorax Clinic Heidelberg, Heidelberg, Germany

**Keywords:** Cancer, Oncology

## Abstract

PIK3CA mutations are common in HR+/HER2− metastatic breast cancer and guide the use of PI3K-targeted therapies. This expert consensus provides recommendations on timing, methods, and interpretation of PIK3CA testing, emphasizing early assessment at metastatic diagnosis, preferred use of NGS panels, and standardized pre-analytical workflows. Reflex and dual-sample strategies can reduce delays and failures. Implementation requires coordinated logistics, education across the diagnostic chain, and equitable access to ensure timely, personalized treatment.

## Introduction

Although metastatic breast cancer (mBC) remains incurable, advances in systemic therapy have significantly improved survival, particularly in the hormone receptor-positive (HR+)/human epidermal growth factor receptor 2–negative (HER2-) subtype. Over the past decade, the addition of cyclin-dependent kinase 4/6 (CDK4/6) inhibitors to endocrine therapy has redefined the standard of the first-line (1L) standard of care, consistently improving progression-free survival (PFS) and, increasingly, overall survival (OS)^1–3^. Recent international guidelines position these combinations as the preferred frontline approach in eligible patients^4,5^.

Nonetheless, most patients progress within 12–36 months of endocrine-based therapy, depending on treatment line and resistance mechanisms^3^. As resistance emerges, therapeutic decisions become more complex. Beyond CDK4/6 inhibitors, subsequent options include phosphoinositide 3-kinase (PI3K) and mechanistic target of rapamycin (mTOR) inhibitors, selective estrogen receptor degraders (SERDs), poly(ADP-ribose) polymerase (PARP) inhibitors in germline *BRCA* mutation carriers, antibody–drug conjugates (ADCs), and cytotoxic chemotherapy. Many of these agents were developed before widespread CDK4/6 use, leaving sequencing strategies uncertain^6,7^.

**Fig. 1:**
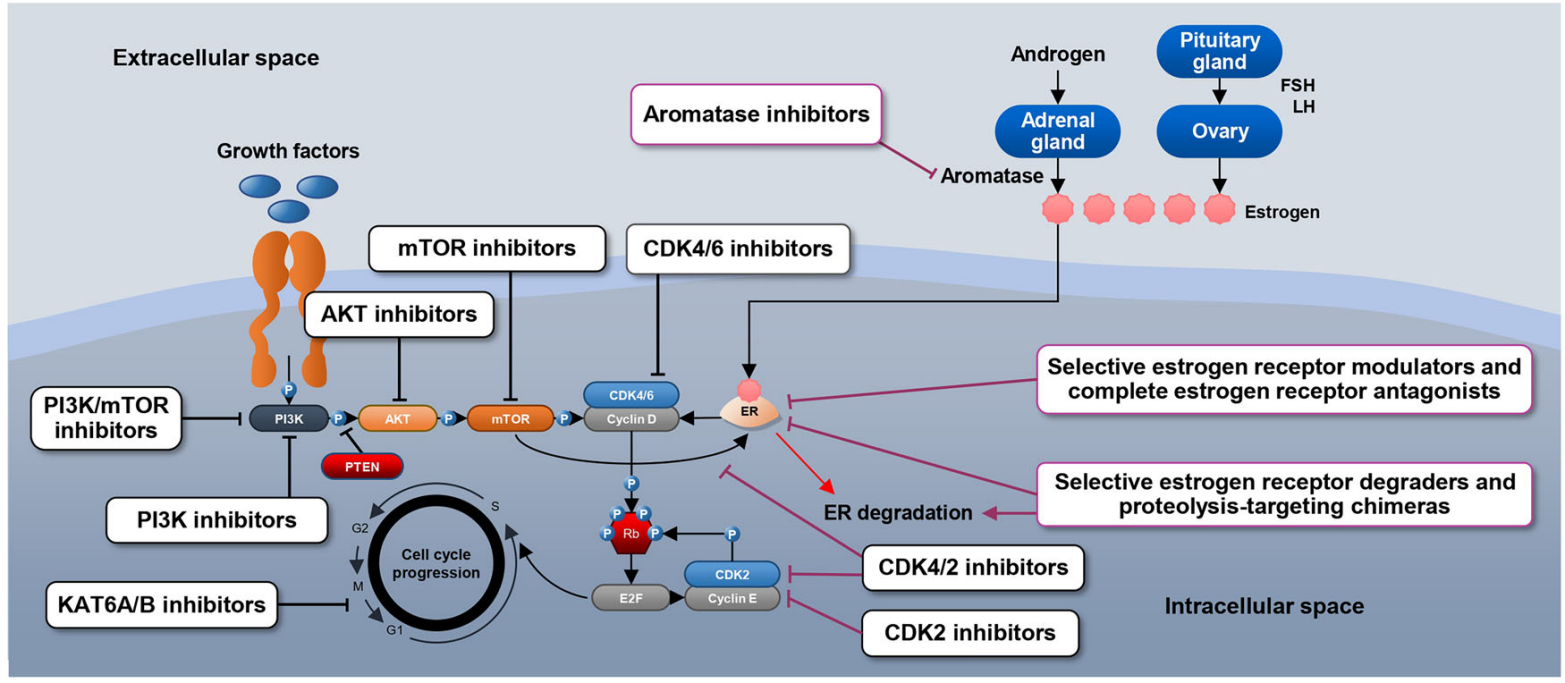
Therapeutic families currently available or under investigation to target the main molecular drivers in HR *+* /HER-mBC. BC breast cancer, CDK cyclin-dependent kinase, ER estrogen receptor, FSH follicle-stimulating hormone, HR+ hormone receptor-positive, LH luteinising hormone, P phosphate.

In parallel, molecular profiling has reshaped luminal mBC management. Genomic testing is now essential for guiding personalized treatment. Deregulation of the PI3K–AKT–mTOR pathway is a central oncogenic mechanism in HR+/HER2− disease and a key driver of endocrine resistance, providing a biological rationale for early detection of potentially actionable alterations (Fig. 1)^8–11^. *PIK3CA* mutation status has become therapeutically relevant, supported by pivotal trials such as SOLAR-1 and BYLieve, in addition to downstream alterations in *PTEN* and *AKT1*, which became targetable recently^12,13^. Moreover, testing for *ESR1* and germline *BRCA1/2* and *PALB2* may enable targeted therapy as well^14–16^.

*PIK3CA* mutations occur in ~40% of HR+/HER2− breast cancers and are directly linked to therapeutic decisions through the use of PI3Kα inhibitors. They predict sensitivity to PI3Kα inhibitors, which have shown clinical efficacy in *PIK3CA*-mutant tumors^17,18^. Recent data suggest a higher prevalence in patients with early progression (≤12 months), reinforcing their prognostic as well as their therapeutic relevance^19^. Accordingly, *PIK3CA* testing is now the standard of care^18^.

Alterations downstream of PI3K, such as *AKT1* mutations and *PTEN* loss, mediate endocrine resistance and were prospectively included as biomarkers for capivasertib, which led to regulatory approval. Although benefit was observed regardless of biomarker status, the current label requires the presence of at least one of these alterations^17^. *ESR1* mutations, in contrast, typically arise after aromatase inhibitor (AI) exposure, leading to constitutive receptor activation and poor response to further endocrine therapy. Circulating tumor DNA (ctDNA)-based *ESR1* testing increasingly informs treatment, including AI-to-SERD switches prior to clinical progression, as explored in SERENA-6^17,18,20^.

Germline *BRCA1/2* and *PALB2* alterations, though more frequent in triple-negative disease, are also found in luminal cancers and predict benefit from PARP inhibitors now approved in HER2-negative carriers^18^.

Collectively, these biomarkers illustrate the molecular complexity of HR+/HER2− mBC and support individualized strategies to overcome resistance, delay chemotherapy, and improve outcomes^17,18,21^.

The rationale for *PIK3CA* testing is twofold: to capture endocrine-resistant disease and to direct patients toward tailored therapy. PI3K/AKT/mTOR pathway activation, most often through *PIK3CA* mutations, drives resistance by bypassing estrogen receptor blockade^18,22^. These alterations bypass estrogen receptor blockade and promote tumor proliferation and survival, limiting the efficacy of standard endocrine therapies^17^. Early identification enables the timely use of targeted agents, avoiding ineffective sequences. *PIK3CA* mutations are also predictive biomarkers for PI3K-targeted therapies. SOLAR-1, the first phase III trial leading to approval, showed alpelisib plus fulvestrant significantly prolonged PFS (11.0 vs. 5.7 months) in *PIK3CA*-mutant disease^12^. More recently, the INAVO120 trial confirmed superior PFS with inavolisib—a potent PI3Kα inhibitor and degrader—combined with palbociclib and fulvestrant in the 1L setting, highlighting its potential in patients with high clinical need^23^.

Beyond PIK3CA, CAPItello-291 showed that biomarker-positive patients (including *PIK3CA, AKT1* and *PTEN*) treated with the AKT inhibitor capivasertib plus endocrine therapy achieved a median PFS of 7.3 vs. 3.1 months with endocrine therapy alone, with similar hazard ratios in the overall population^16^. The NATALEE clinical trial further suggests that some high-risk HR+/HER2− patients recur despite extended endocrine therapy and CDK4/6 inhibition, representing a potential target population for PI3K inhibition in the metastatic setting^24^.

This expert opinion manuscript provides guidance on implementing *PIK3CA* mutation testing in HR+/HER2− mBC in the context of recent advances, including the recent European Medicines Agency approval of inavolisib for PIK3CA-mutated, ER+/HER2− locally advanced or metastatic breast cancer following recurrence on or within 12 months of completing adjuvant endocrine therapy. It addresses when, how, and for whom testing should be performed, integrating evidence from literature with insights from an international panel of oncologists, pathologists, and molecular diagnosticians.

## Timing and candidates for testing (WHEN/WHOM)

Determining the optimal timing for *PIK3CA* testing is critical for ensuring that molecular data can be effectively integrated into treatment decision-making. In patients with HR+/HER2− metastatic breast cancer, current clinical guidelines and expert consensus converge on the need for early testing—ideally at the time of metastatic diagnosis—before initiating 1L systemic therapy^1,25^.

Early testing enables timely identification of actionable alterations such as *PIK3CA*, *PALB2*, or *BRCA1/2* mutations, which may influence therapeutic selection from the outset. This is not only based on actionability but also facilitates the integration of prognostic odds within a molecular-built and clinically meaningful portrait of the disease as early as possible. Moreover, with the approval of inavolisib in first-line treatment for endocrine-resistant disease, testing before or at the onset of mBC treatment has become mandatory if access to the drug is available. Deferring testing until progression can result in suboptimal use of targeted therapies and decisions based on outdated tissue samples, which may no longer reflect the current mutational profile of the tumor^26,27^.

**Fig. 2:**
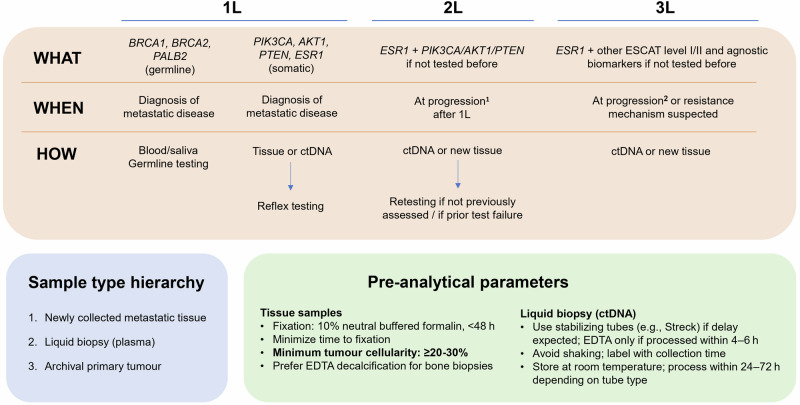
Recommended testing workflow across treatment lines in HR *+* /HER-mBC. 1L first-line, 2L second-line, 3L third-line, ctDNA circulating tumor DNA, EDTA ethylenediaminetetraacetic acid. ^1^If not previously assessed or if baseline testing failed/non-evaluable. ^2^ESCAT level I biomarkers refer to molecular alterations with the highest level of clinical actionability according to the ESMO Scale for Clinical Actionability of molecular Targets (ESCAT), i.e., supported by high-level prospective clinical evidence with a matched targeted therapy. ESCAT level II biomarkers refer to alterations with emerging or context-dependent clinical actionability supported by clinical evidence, but with a lower level of evidence than ESCAT level I (e.g., derived from non-biomarker-selected trials, subgroup analyses, or early-phase/basket studies), and typically considered on a case-by-case basis. In this workflow, examples of ESCAT level I include PIK3CA, AKT1, PTEN, and ESR1 alterations, together with *PALB2* and BRCA1/2 germline status; additional ESCAT level I/II alterations and agnostic biomarkers may be considered (if not tested before) according to local availability and molecular tumor board recommendations.

A tiered testing strategy has been proposed to reconcile clinical relevance when real-world constraints are present. In this model, mutations with established therapeutic implications—such as *PIK3CA* and *BRCA1/2*—are prioritized before 1L therapy (testing performed on the diagnostic biopsy of metastatic disease, Fig. 2)^1,25^. Other biomarkers, such as *ESR1*, which are often acquired during endocrine therapy, might be evaluated later, at the time of disease progression (Fig. 2). At first progression on endocrine-based therapy, plasma ctDNA is a pragmatic option to expedite biomarker assessment, particularly for *ESR1*. In addition, if baseline profiling did not include PIK3CA/AKT1/PTEN or yielded a non-evaluable result, ctDNA testing at progression can recover these actionable alterations in time to inform second-line decisions.

Conversely, if high-quality baseline testing was successfully completed and was negative for PIK3CA/AKT1/PTEN, routine re-testing at first progression is generally not recommended, given the lower likelihood of conversion from negative to positive compared with *ESR1*, which is frequently acquired under endocrine selective pressure and may warrant serial testing across lines. This approach reflects not only biological dynamics but can also help in terms of logistical and economic considerations^28^. This does not apply in case Institutions are routinely using panels with a high reference range, i.e., panels that include all genes that are relevant for breast cancer and other malignancies. In this scenario, one test—typically on a biopsy of metastatic disease—is offering complete results covering all genes of interest and agnostic biomarkers (including complex biomarkers such as tumor mutational burden and microsatellite instability). Nevertheless, liquid biopsies should be considered to best capture and monitor the emergence of resistance mutations over time (Fig. 2).

Turnaround time after requesting a test is also a practical challenge. While less than two weeks is considered ideal, especially when testing informs 1L treatment initiation, such timelines may not be feasible in many public or resource-constrained settings. Delays beyond this window risk rendering results irrelevant for immediate clinical decisions. In some health systems, the timing of testing can also impact reimbursement eligibility, further complicating access^29^.

As a consensus approach addressing both clinical utility and systemic limitations, *PIK3CA* testing should be offered at the time of diagnosis of advanced or metastatic HR+/HER2− disease^1^, and results should be available within 2 to 3 weeks, regardless of whether tissue or liquid biopsy is used^30^. This timeframe, as a benchmark, can be met across healthcare systems, balancing feasibility and urgency, maintaining the opportunity to inform treatment without the use of a bridging therapy for the vast majority of front-line patients. Institutional adoption of reflex testing and streamlined logistics is encouraged to approach this target and improve clinical integration.

### Reflex testing

To accelerate access to clinically actionable results, reflex testing has been proposed as a pragmatic solution in newly diagnosed HR+/HER2− metastatic or advanced breast cancer. This approach involves pre-defined workflows, often defined by standard operating procedures (SOP), that allow pathologists or molecular laboratories to automatically initiate biomarker testing—such as *PIK3CA* testing—once specific clinical or histological criteria are met. While not yet widely implemented, reflex testing could reduce delays and ensure the timely initiation of appropriate targeted therapies. Integration of reflex testing into multidisciplinary team discussions can further optimize patient management, ensuring that molecular results are promptly reviewed alongside other clinical information to guide treatment decisions^31,32^.

## Testing methods and pre-analytics (HOW)

### Sample hierarchy and pre-analytical conditions

The reliability of *PIK3CA* molecular testing in HR+/HER2− mBC depends critically on the type and quality of the biological sample, the preferred hierarchy being as follows: newly diagnosed metastatic tissue as the first option, followed by liquid biopsy and archival primary tumor (Fig. 2)^27,33^. As *PIK3CA* is a truncal mutation, archival primary tumor is also an option, although it may not reflect the current mutational landscape due to spatial and temporal heterogeneity^27,33^. Temporal heterogeneity, clonal evolution, and treatment-induced selection pressure can all contribute to discrepancies between archival and current biopsy findings.^26,27^. To ensure reliable variant detection in tissue samples, a minimum tumor cellularity of 20–30% is recommended^34,35^. Below this threshold, especially in formalin-fixed paraffin-embedded (FFPE) tissue, there is an increased risk of false-negative results due to low mutant allele frequency^36^.

Particular caution is needed in bone biopsies, which are frequent in HR+/HER2− mBC. Ethylenediaminetetraacetic acid (EDTA)-based decalcification should be used instead of acid-based protocols to preserve nucleic acid integrity, which is essential for both DNA and RNA-based assays^37^.

Proper fixation protocols are essential for all tissue samples. Standard practice recommends using 10% neutral buffered formalin, with fixation times not exceeding 24–48 h^38^. Deviations from these protocols can lead to nucleic acid degradation and compromised sequencing performance^39^.

For liquid biopsies, the pre-analytical phase is equally critical and fundamentally different from that of FFPE samples. ctDNA is highly sensitive to processing delays, and improper handling can cause leukocyte lysis, leading to contamination with germline DNA^40^. Blood samples should ideally be collected in specialized tubes (e.g., Streck) that stabilize cell-free DNA (cfDNA) for up to 7 days at room temperature, especially if immediate processing is not possible^41^. Tubes must be handled gently, avoiding shaking, and labeled with collection time to ensure traceability^42,43^.

Finally, coordination between clinicians, nurses, pathologists, and laboratory teams is crucial. Clear guidance should be established for sample collection, tumor content estimation, tube selection, and transport protocols to reduce the risk of non-evaluable samples and ensure molecular testing is both robust and reproducible^44^.

### Sample collection and logistics

Optimizing sample collection and handling is essential to ensure the success of *PIK3CA* testing in advanced and metastatic breast cancer. A dual-sample strategy—collecting both tissue and liquid biopsy at diagnosis—is perceived as ideal by the authors and is increasingly adopted to minimize diagnostic delays and reduce the risk of sample failure. This approach allows complementary molecular information to be derived from both sources, especially in heterogeneous disease settings^45^.

The type of sample used remains a topic of ongoing debate. Tissue biopsy allows for a morphology-based approach with details on tumor morphology and cellularity, and holds a more solid preanalytical workflow. However, ctDNA offers a minimally invasive alternative that can better reflect tumor heterogeneity across metastatic sites and can be informative in cases where recent tissue is unavailable or inadequate^25,27,28,46–48^. Recent guidelines and studies recommend a stepwise approach in which ctDNA may be used initially, with reflex tissue testing advised in case of negative or inconclusive plasma results^32,49^.

To implement this strategy effectively, clinical workflows may benefit from a structured decision-making algorithm that takes into account:

1. Clinical need, particularly when rapid therapeutic decisions are required^50^.
2. Turnaround times, which vary depending on the testing platform/testing technique and sample type^50,51^.
3. Sample availability, especially when recent metastatic tissue is not accessible^52^.
4. Cost-effectiveness and feasibility in different institutional settings as well as healthcare systems^28,53^.

Storage and transport conditions must follow manufacturer guidelines. Samples should remain at room temperature and be processed within 24–72 h, depending on tube specifications^54^. Improper handling or extended delays can lead to cfDNA degradation and false-negative results^42^.

Preanalytical workflows in the context of liquid biopsies are less known and education in this respect should be performed: training of involved personnel is essential to ensure protocol adherence across all stages—from collection to laboratory reception. Nurses, radiologists, and transport staff should be familiar with proper tube usage, labeling, fixation protocols, and storage procedures^55^. Specifically, the use of EDTA over acid decalcifiers in bone biopsies is crucial to maintain nucleic acid integrity, as discussed in prior sections^37^.

The recommended testing workflow and pre-analytical requirements are summarized in Fig. 2, which outlines when and how to test across treatment lines, preferred sample types, and critical handling considerations to ensure reliability and clinical utility.

### Testing platforms

For the experts of this consensus, next-generation sequencing (NGS) by targeted panels should be the standard method for biomarker testing in HR+/HER2− metastatic breast cancer. Targeted NGS panels enable the simultaneous detection of multiple genomic alterations, improving efficiency. Whenever large panels (100–500 genes or more) are used, a comprehensive genomic profiling can be ensured from limited tissue or liquid biopsy samples^56,57^. Importantly, the panel recommended that any testing platform should, at a minimum, include European Society for Medical Oncology (ESMO) Scale for Clinical Actionability of molecular Targets (ESCAT) Level I alterations, such as *PIK3CA*, *ESR1*, *BRCA1/2*, *PTEN*, and *AKT1* mutations. These alterations have established clinical utility and are directly linked to the availability of targeted therapies^58^.

Two main NGS methodologies are widely used:

- Amplicon-based panels are common due to their lower DNA requirements, faster processing times, and reduced cost^59,60^. However, they offer limited genomic coverage and lower sensitivity for detecting structural variants or copy number alterations^61^.
- Hybrid capture-based platforms provide broader coverage and improved sensitivity for low-frequency mutations and complex genomic events^59,60,62^, though they require more input material, longer turnaround, and greater laboratory infrastructure^62^.

When NGS is not available due to infrastructure limitations, cost, or restricted access, quantitative PCR (qPCR) represents a valid alternative for *PIK3CA* testing. qPCR offers several practical advantages: it is a rapid, low-cost, and user-friendly technique that can be readily implemented in routine hospital settings. In countries or institutions where NGS is not widely accessible, qPCR-based assays allow clinicians to identify actionable PIK3CA mutations and guide treatment decisions, helping to bridge the gap in molecular diagnostics and support broader patient access to targeted therapies^5^.

The choice of platform should be guided by clinical needs, technical performance, sample quality, and institutional resources.

Although access to targeted therapies remains limited in some healthcare systems, the authors propose a testing strategy (Fig. 1) that prioritizes the best available evidence to maximize clinical benefit while conserving resources. Reflecting evidence for the pronounced benefit of the use of PARP inhibitors as early as possible, biomarkers known to be stable over time should be spared from retesting. Along the same line, therapy informing, but a frequently acquired *ESR1* mutation may require serial testing upon progression^60,63^.

## Interpretation and reporting (ESMO alignment)

Standardized reporting is essential to ensure genomic testing is interpretable across multidisciplinary teams. Reports should follow ESMO recommendations on variant annotation and classification, enabling both oncologists and pathologists to distinguish between clinically actionable mutations and variants of uncertain significance. Consistent use of standardized nomenclature (e.g., Human Genome Variation Society) and inclusion of the ESMO Scale for Clinical Actionability of molecular Targets (ESCAT) levels of actionability helps contextualize molecular findings within therapeutic frameworks. According to the ESCAT, Level 1 alterations are those with the highest level of evidence, supported by prospective clinical trials showing improved outcomes with a targeted therapy. In this setting, ESCAT 1A alterations are considered directly actionable with approved PI3Kα inhibitors^1^.

To enable a detailed discussion of the functional significance of *PIK3CA* alterations, actionability and ultimately, prioritization of therapies, the authors recommend making use of molecular tumor boards (MTB), wherever available. These boards co-evolved with a growing landscape of complex biomarkers, providing valuable expertise for interpreting variants and guiding off-label or trial-based treatment decisions^64^.

The limitations of ctDNA testing were acknowledged previously in this article. In cases where liquid biopsy yields a negative result despite clinical suspicion of progression or resistance, repeat testing or confirmation with tissue biopsy should be considered, in line with current ESMO guidance^1^.

The panel emphasized that not all mutations detected by NGS are relevant for treatment selection. It is therefore essential to distinguish between variant annotation (a technical description) and clinical actionability (the implication for therapy) (Table 1)^1,22,63^. Reports should clearly indicate whether a variant is actionable, prognostic, or incidental^63^.

**Table 1:** Clinical classification of PIK3CA mutations based on ESCAT level and therapeutic relevance in HR+/HER2*−* metastatic breast cancer^1,16,22,23,63,80–86^

Mutation	Exon	Evidence level	Clinical actionability
H1047R	20	1 – ESCAT 1-A	Actionable-Eligible for inavolisib/alpelisib/capivasertib
H1047L	20	1 – ESCAT 1-A	Actionable-Eligible for inavolisib/alpelisib/capivasertib
E545K	9	1 – ESCAT 1-A	Actionable-Eligible for inavolisib/alpelisib/capivasertib
E542K	9	1 – ESCAT 1-A	Actionable-Eligible for inavolisib/alpelisib/capivasertib
E545A	9	1 – ESCAT 1-A	Actionable-Eligible for inavolisib/alpelisib/capivasertib
E545G	9	1	Eligible for inavolisib/alpelisib/capivasertib
Q546R	9	1	Eligible for inavolisib/alpelisib/capivasertib
C420R	7	1	Eligible for inavolisib/alpelisib/capivasertib
Q546K	9	Preclinical	Eligible for inavolisib/capivasertib
N345K	4	Preclinical	Eligible for inavolisib/capivasertib
G1049R /S	20	Preclinical	Eligible for inavolisib/capivasertib (for capivasertib G1049R only)
E726K	13	Co-mutated	Limited evidence
G118D	2	Unknown	Eligible for inavolisib
E453K	7	Unknown	Eligible for inavolisib
M1043I/T/V	20	Unknown	Eligible for inavolisib/capivasertib (for capivasertib M1043I/V only)
N1068K	20	Unknown	Uncertain clinical significance
E81K	2	Unknown	Uncertain clinical significance
H1047D/N/P/Q/T/I	20	Unknown	Eligible for inavolisib
H1047Y	20	Unknown	Eligible for alpelisib/capivasertib
E110del	2	Unknown	Uncertain clinical significance
Q546E/H/L/P	9	Unknown	Eligible for inavolisib/alpelisib (for alpelisib Q546E only)/capivasertib (for capivasertib Q546E/p only)
R88Q	1	Unknown	Eligible for inavolisib/capivasertib
G106A/D/R/S/V	1	Unknown	Eligible for inavolisib
K111N/R/E	1	Unknown	Eligible for inavolisib
N345D/H/I/S/T/Y	4	Unknown	Eligible for inavolisib
E453A/D/G/Q/V	7	Unknown	Eligible for inavolisib
E545D/Q/V	9	Unknown	Eligible for inavolisib/alpelisib (for alpelisib E545D [1635G > T only])
E542A/D/Q/R/V	9	Unknown	Eligible for inavolisib/capivasertib (for capivasertib E545A/D/Q)

Interpretation of variant allele frequency (VAF) remains a challenge due to the lack of universally accepted thresholds. Technical guidance suggests that reliable detection of variants at or above 3% VAF requires a sequencing depth of at least 1650 reads with a minimum of 30 mutated reads, which minimizes the risk of false-negative or false-positive results^65^. Variants detected below this threshold should be interpreted with caution and, where possible, confirmed by orthogonal methods or replicate analyses. Reports should also specify key technical parameters—including assay error rate, DNA quality and quantity, minimum coverage, and limit of detection—to provide clinicians with sufficient context for interpreting VAF results^65^.

## Quality standards and implementation (QUALITY)

Ensuring the clinical utility of *PIK3CA* mutation testing in HR+/HER2− metastatic breast cancer requires robust quality standards across all stages of the diagnostic pathway. The success of molecular profiling should not be assessed solely by analytical performance but also by its impact on clinical outcomes. Incorporating biomarker-derived indicators into Clinical Outcome Measures (COM) frameworks—such as time to treatment initiation, access to targeted therapies, and progression-free survival in real-world settings—is recommended to evaluate the true effectiveness of implementation^66^.

Testing failures, often due to poor DNA quality, low tumor content, or suboptimal pre-analytical conditions, must be anticipated and minimized. In the event of sample failure, selection of alternative specimens should be tailored to the clinical question and the alteration being assessed. For baseline genomic profiling at metastatic diagnosis (including *PIK3CA* mutations and other established actionable biomarkers), the recommended hierarchy of alternative samples is: (a) metastatic tissue biopsy, (b) liquid biopsy (ctDNA), and (c) archival primary tumor tissue. This order reflects both the biological relevance of the sample and its likelihood of yielding interpretable genomic results. By contrast, whenever testing is aimed at identifying acquired endocrine-resistance alterations (e.g., *ESR1* mutations) at disease progression, plasma ctDNA should be prioritized when feasible. If ctDNA is unavailable or yields a negative/inconclusive result in a context of high clinical suspicion, a new metastatic tissue biopsy is recommended as back-up, whereas archival primary tumor tissue is generally not appropriate to rule out acquired endocrine-resistance genetic alterations^67^.

Validated protocols should be applied consistently at every step—from sample collection to laboratory processing—to reduce the risk of inconclusive or non-evaluable results. Standard operating procedures must define fixation methods, exposure times, transport conditions, and minimum tumor cellularity thresholds to ensure reliability^68^.

While addressed elsewhere in this article, it is worth reiterating that strategies such as simultaneous collection of tissue and blood samples, and reflex testing, are highly recommended to reduce turnaround times and minimize delays^69^.

Pre-analytical quality control is particularly important in bone metastasis biopsies. The use of EDTA-based decalcification is preferred over acid-based agents to preserve nucleic acid integrity and avoid sequencing artifacts. These requirements should be clearly communicated and incorporated into institutional workflows to avoid preventable test failures^37^.

Combined with strong coordination between clinical and laboratory teams, these measures help ensure that molecular results are available at the point of therapeutic decision-making.

## Barriers, equity, and access considerations

Despite growing evidence supporting the clinical utility of molecular testing in HR+/HER2− metastatic and advanced breast cancer, significant disparities in access persist across regions and institutions^70^. In many healthcare systems, the availability of biomarker testing is closely tied to local infrastructure, reimbursement pathways, and clinician awareness—factors that often differ widely between urban and underserved settings^70,71^.

Limited access to NGS or validated liquid biopsy platforms can result in underutilization of targeted therapies, even in eligible patients. This gap is further exacerbated when testing protocols are not standardized or widely disseminated, leading to variability in clinical practice and potential inequities in treatment outcomes^72^. This gap could be partially addressed by implementing alternative techniques validated for *PIK3CA* testing, such as PCR-based assays, which are more accessible, cost-effective, and feasible in settings with limited molecular infrastructure^5^.

Raising awareness among clinicians—particularly those practicing in resource-limited or peripheral centers—was identified as a key strategy to close this gap^73^. Clear clinical guidelines, diagnostic algorithms, and educational efforts are essential to ensure that molecular testing becomes a consistent part of the care pathway, rather than an optional or delayed step. Without such efforts, patients may remain untested simply due to a lack of local protocols, insufficient logistical support, or limited genomic literacy among providers^73,74^.

Addressing these barriers will require a combination of policy, education, and system-level investment to ensure that genomic advances translate into equitable care across diverse healthcare settings.

## Recommendations for practice and policy

Looking ahead, the forthcoming integration of PI3K inhibitors into routine clinical practice reinforces the urgency of optimizing testing workflows for HR+/HER2− metastatic breast cancer. Early identification of *PIK3CA* mutations will be essential to ensure timely patient access to this and other emerging therapies, avoiding treatment delays linked to diagnostic bottlenecks^63^.

One of the strongest recommendations is the need to invest in education across the diagnostic chain^75^, ensuring that every stakeholder involved in the testing process is aware of their role and the impact of their actions on test success and turnaround time:

- Oncologists should receive focused training on how to incorporate biomarker testing into treatment decision-making, and how to identify which patients are eligible within their specific practice environment^76^.
- Surgeons and interventional radiologists must be trained to collect adequate tissue samples, minimize fixation delays, and apply proper labeling protocols—particularly in bone biopsies, where decalcification methods can affect test viability^37,77^.
- Nurses, care assistants, phlebotomists and transport personnel should be instructed on tube handling, labeling, and the importance of avoiding physical agitation (e.g., not shaking blood tubes), which may degrade sample quality before analysis^78^.
- Pathology laboratories and logistics teams are encouraged to develop or refine traceability systems, improve internal communication, and coordinate test flows across departments to minimize delays and reduce the risk of failure due to pre-analytical errors^79^.

## Conclusions

The integration of *PIK3CA* testing into the clinical management of HR+/HER2− metastatic breast cancer represents a critical step toward personalized therapy as early as the first line of mBC treatment. Testing for *PIK3CA* mutations is no longer optional—it is essential for identifying patients who may benefit from PI3Kα inhibitors and informing therapeutic sequencing in an increasingly complex landscape.

To ensure clinical utility, testing must be timely, technically robust, and logistically feasible. Early testing at the time of metastatic diagnosis is recommended to guide 1L or early-line decisions, and reflex testing workflows—along with dual-sample strategies using tissue and liquid biopsy—should be implemented to minimize delays and sample failures. NGS platforms are preferred due to their ability to capture a wide range of actionable alterations, including uncommon or less frequent PIK3CA variants. PCR platforms should be considered whenever NGS testing is not available.

The reliability of results hinges on standardized pre-analytical protocols and cross-disciplinary coordination across collection, processing, and reporting steps. Institutions must define clear quality criteria, including minimum tumor cellularity and fixation standards, and prioritize training across the diagnostic chain to improve turnaround times and reduce variability. Beyond technical aspects, testing performance should be evaluated in terms of real-world clinical outcomes. Embedding biomarker data into COM frameworks—such as time to treatment initiation and access to targeted therapies—will help quantify the real impact of implementation.

As PI3K inhibitors become part of routine clinical care, optimizing biomarker testing will be essential to ensure that patients with *PIK3CA*-mutant tumors are identified early and treated effectively, regardless of institutional setting or resource level. The recommendations outlined in this manuscript provide a practical roadmap to support the equitable and consistent adoption of *PIK3CA* testing in locally advanced and metastatic breast cancer.

## Data Availability

No datasets were generated or analyzed during the current study.
